# 

**Published:** 1891-11

**Authors:** 


### TABLE OF CONTENTS.

ORIGINAL COMMUNICATIONS. PAGE

A Few Words in Regard to the Sterilization of Instruments. By Harold C. Ernst, M.D. . . . . . 741

Histological Methods and Results. By L. C. Ingersoll, Keokuk, Iowa . 748

Alveolotomy. By G. S. Junkerman, M.D., D.D.S., Cincinnati, Ohio . . 760

A New Method of Running the Suspension Dental-Engine by the Electric Current. By T. A. Fletcher, D.D.S., New York . . . . . 762

REPORTS OF SOCIETY MEETINGS.

American Dental Association . . . . . 764

Harvard Odontological Society . . . . . 779

Odontological Society of Pennsylvania . . . . . 786

EDITORIAL.

The Dental Protective Association . . . . . 798

The "Dental Advertiser" and its New Editor . . . . . 800

Bibliography . . . . . 800

OBITUARY.

C. A. Kingsbury, M.D., D.D.S. . . . . . 801

Dr. David Roberts . . . . . 803

CURRENT NEWS.

American Academy of Dental Science . . . . . 804

Anniversary Meeting of the First District Dental Society . . . . . 804

